# Supplementary material for: Evaluation of COVID-19 antigen rapid diagnostic tests for self-testing in Lesotho and Zambia
Source: PLoS One. 2024 Feb 29;19(2):e0280105. doi: 10.1371/journal.pone.0280105 (PMC10903820; doi:10.1371/journal.pone.0280105)
Supplement: S1 Appendix — (PDF) [file pone.0280105.s001.pdf]

EN REF Q-NCOV-03G

STANDARD Q
COVID-19 Ag Home Test

STANDARD™ Q COVID-19 Ag Home Test

Video Guide

SELF-DIAGNOSTIC TEST
Fast results in 15 minutes | Nasal Test

COVID-19 Antigen Test
Instructions for Use

SD BIOSENSOR

Product name: STANDARD™ Q COVID-19 Ag Home Test
Model name: STANDARD™ Q COVID-19 Ag Home Test (Q-NCOV-03G)
Intended use: STANDARD Q COVID-19 Ag Home Test is a rapid chromatographic immunoassay for the qualitative detection of SARS-CoV-2 nucleocapsid antigen present in human nasal sample. It provides only an initial screening test result. The result of this test should not be the sole basis for the diagnosis; confirmatory testing is required. For serial testing programs, additional confirmatory testing with a molecular test for negative results may be necessary, if there is a high likelihood of COVID-19, such as, an individual with a close contact with COVID-19 or with suspected exposure to COVID19 or in communities with high prevalence of infection. Additional confirmatory testing with a molecular test for positive results may also be necessary, if there is a low likelihood of COVID-19, such as in individuals without known exposures to COVID-19 or residing in communities with low prevalence of infection.

[When to use this kit]
Do use
- If you want to diagnosis current COVID-19 infection.
- If you are concerned that you may have COVID-19.

Do not use
- If you can't collect sample in the recommended way.
- If you are prone to nosebleeds.

EXPLANATION AND SUMMARY
■ Introduction
Coronavirus is a single-stranded positive-sense RNA virus with an envelope of about 80 to 120 nm in diameter. Its genetic material is the largest of all RNA viruses and is an important pathogen of many domestic animals, pets, and human diseases. It can cause a variety of acute and chronic diseases. Common symptoms in people infected with coronavirus include respiratory symptoms, fever, cough, shortness of breath, and dyspnea. In severe cases, infection can cause pneumonia, severe acute respiratory syndrome, kidney failure, and even death. "2019-nCoV" was discovered because of Wuhan Viral Pneumonia cases in 2019 and on January 12, 2020, World Health Organization (WHO) confirmed that it can cause colds like Severe Acute Respiratory Syndrome (SARS), and Middle East Respiratory Syndrome (MERS).

■ Test Principle
STANDARD Q COVID-19 Ag Home Test has two pre-coated lines, "C" Control line, "T" Test line on the surface of the nitrocellulose membrane. Both the control line and test line in the result window are not visible before applying any sample. Mouse monoclonal anti-SARS-CoV-2 antibody is coated on the test line region and mouse monoclonal anti-Chicken IgY antibody is coated on the control line region. Mouse monoclonal anti-SARS-CoV-2 antibody conjugated with color particles are used as detectors for SARS-CoV-2 antigen device. During the test, SARS-CoV-2 antigen in the sample interact with monoclonal anti-SARS-CoV-2 antibody conjugated with color particles making antigen-antibody color particle complex. This complex migrates on the membrane via capillary action until the test line, where it will be captured by the mouse monoclonal anti-SARS-CoV-2 antibody. A colored test line would be visible in the result window if SARS-CoV-2 antigens are present in the sample. The intensity of colored test line will vary depending upon the amount of SARS-CoV-2 antigen present in the sample. If SARS-CoV-2 antigens are not present in the sample, then no color appears in the test line. The control line is used for procedural control, and should always appear if the test procedure is performed properly and the test reagents of the control line are working.

CONTENTS
Test device, Solution tube & Nozzle cap, Sterile swab, Instructions for use & Quick reference instruction

INSTRUCTION FOR USE
■ Sample Collection
[Nasal swab]
1. Open the seal of solution tube and set the solution tube on the stand hole of the package.
2. Open the sterile swab pouch.
3. Tilt head back 70 degrees.
4. While rotating the swab, insert it less than one inch (about 1.5 cm) into nostril.
5. Rotate the swab 10 times against nasal wall.
6. Repeat in other nostril using the same swab.

■ Preparation
1. Carefully read instructions for using the STANDARD Q COVID-19 Ag Home Test.
2. Check the expiry date at the back of the foil pouch. Do not use the kit, if expiry date has passed.
3. Check the test device and the desiccant pack in the foil pouch.

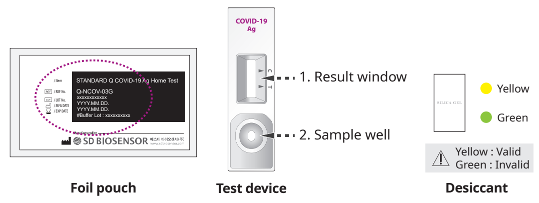

■ Test Procedure
1. Insert the swab into an solution tube. While squeezing the solution tube, stir the swab more than 10 times.
2. Remove the swab while squeezing the sides of the tube to extract the liquid from the swab.
3. Press the nozzle cap tightly onto the tube.
4. Apply 4 drops of extracted sample to the sample well of the test device.
5. Read the test result in 15 minutes.

⚠ Do not read test results after 30 minutes. It may give false results.

INTERPRETATION OF TEST RESULT

Results	Example	Interpretation of results
Negative	C T	Negative result: A colored band will appear only control line(C) on the result window.
Positive	C T / C T	Positive result: Colored bands will appear both control line(C) and test line(T) on the result window.
Re-test	C T / C T	Retest: If the control line(C) is not appeared in the result window, it is an invalid result. Retest using a new sample and device.

* Positive results should be considered in conjunction with the clinical history and other data available.
* Even if the control line is faint, or the test line isn't uniform, the test should be considered to be performed properly and the test result should be interpreted as a positive result.

KIT STORAGE AND STABILITY
Store the kit at 2-30°C / 36-86°F out of direct sunlight. Kit components can be stably used until the expiration date printed on the external box. Do not store the kit frozen.

PRECAUTIONS FOR USE
1. This product should be used for in vitro diagnosis.
2. COVID-19 self-diagnosis test is a novel coronavirus antigens diagnosis medical device using the nasal.
3. Since the SARS-CoV-2 antigen rapid test is a screening test, it is recommended to conduct the test periodically.
4. Before testing, read the instruction for use and follow the test procedure.
5. This product has been clinically evaluated on patient sample after the onset of COVID-19 symptoms, and was not clinically evaluated on asymptomatic patients.
6. After testing, wash or disinfect your hands thoroughly with soap and running water.

■ Precautions for Results Review
1. It is not possible to accurately diagnose SARS-CoV-2 infection only with the result of this product. When subject to a thorough examination, the doctor must make the final decision by considering clinical symptoms after performing a confirmation test with an approved RT-PCR product (except for emergency screening).
2. If the concentration of SARS-CoV-2 antigen in the sample is less than the detection limit of the test, or if it is collected or transported improperly, a false negative result may appear. Therefore, the possibility of SARS-CoV-2 infection cannot be eliminated with a negative result.
3. This product only checks the presence of SARS-CoV-2 antigen, and there is no correlation between the strength of the test line and the concentration of SARS-CoV-2 antigen.
4. If there is a mutation in the binding area of the monoclonal antibody contained in this product, the sensitivity may be reduced.
5. This product cannot distinguish between SARS-CoV and SARS-CoV-2 antigens.
6. This product only checks the presence of SARS-CoV-2 antigen, and there is no correlation between the strength (or measured value) of the test line and the concentration of SARS-CoV-2 antigen.
7. Sample collected after 6 days from the onset of symptoms may have false negative results.

AFTER THE TEST
After the test, follow the follow-up instructions below.

■ Check for Positive COVID-19 Result
If you have a positive result, it is very likely that you have COVID-19.

[What you need to do]
1. You should self-isolate to avoid spreading the virus to others. There is a very small chance that this test can give a positive result that is wrong (a false positive result).
2. Consult a healthcare professional as soon as possible. Your healthcare provider will work with you to determine how best to care for you based on the test results along with other factors of your medical history, and your symptoms, possible exposures, and geographic location of places you have recently traveled.

■ Check for Negative COVID-19 Result
A negative test result means that the SARS-CoV-2 antigen that causes COVID-19 was not found in your sample. For COVID-19, a negative test result for a sample collected while a person has symptoms usually means that COVID-19 did not cause your recent illness.

[What you need to do]
Please consult a healthcare professional if you develop symptoms, symptoms persist or become more severe. It is possible for this test to give a negative result that is incorrect (false negative) in some people with COVID-19. This means that you could possibly still have COVID-19 even though the test is negative. If this is the case, your healthcare provider will consider the test result together with all other aspects of your medical history (such as symptoms, duration of illness, possible exposures, and geographical location of places you have recently traveled) in deciding how to care for you. It is important that you work with your healthcare provider to help you understand the next steps you should take.

■ Check for Invalid Result
An invalid test result means that the your test has experienced an error as below.

[The reason for the error occurred]
- When tested without proper understanding of how to use it
- When the insufficient or excessive sample is used
- When the user checks the result at the wrong reading time

[What you need to do]
When your test has experienced an error, you will need to retest with a new test or consult a healthcare professional. Until confirming the new test result, you need to do self-isolate at home. If you have any questions, please contact us to helpline 1-888-885-6121 / email (help.covid19@sdbiosensor.com) and website (www.sdbiosensor.com).

USER SAFETY
[What you need to know]
Older adults and people who have severe underlying medical conditions like heart or lung disease or diabetes seem to be at higher risk for developing more serious complications from COVID-19 illness.

[High risk groups]
- People aged 65 years and older
- People in nursing homes or long-term care facilities
- People of all ages with underlying medical conditions, particularly if they are not well controlled:
  - Cancer
  - Chronic obstructive pulmonary disease
  - Chronic kidney disease
  - Immunocompromise such as those post solid-organ transplant
  - Obesity (BMI >30)
  - Serious heart conditions such as heart failure, coronary artery disease, cardiomyopathies
  - Sickle cell disease
  - Type II Diabetes
  - Immunocompromised
  - Asthma (Moderate-severe)
  - Cerebrovascular disease (affecting blood vessels to the brain)
  - Cystic fibrosis
  - High blood pressure
  - Immunocompromised (weakened immune system) from blood or bone marrow transplants, immune deficiencies, HIV, use of corticosteroids, use of other immune weakening medications
  - Neurologic conditions such as dementia
  - Liver disease
  - Pregnancy
  - Pulmonary fibrosis
  - Smoking
  - Thalassemia
  - Type I Diabetes

[When to seek emergency medical attention]
Look for emergency warning signs* for COVID-19. If someone is showing any of these signs, seek emergency medical care immediately:
- Trouble breathing
- Persistent pain or pressure in the chest
- New confusion
- Inability to wake or stay awake
- Bluish lips or face

*This is not an exhaustive symptom list. Please call your medical provider for any other symptoms that are severe or concerning to you.

Call 911 or call ahead to your local emergency facility:
Notify the operator that you are seeking care for someone who has or may have COVID-19.

FAQS
What are the known and potential risks and benefits of this test?

Potential risks include:
- Possible discomfort during sample collection.
- Possible incorrect test results (see Results section).
  - Potential benefits include:
    The results, along with other information, can help your healthcare provider make informed recommendations about your care.
  - The results of this test may help limit the spread of COVID-19 to your family and others in your community.
  - You have the option to refuse this test. However, your doctor has prescribed this test because they believe it could help with your care.

MORE ABOUT THE TEST
1. This product contains small amounts of animal sourced materials.
2. If you have any questions about the product, please contact your local distributor.
3. You can also visit www.sdbiosensor.com for product demonstrations.

COVID-19 INFORMATION
What is COVID-19?
COVID-19 is caused by the SARS-CoV-2 virus which is a new virus in humans causing a contagious respiratory illness. COVID-19 can present with a mild to severe illness although some people infected with COVID-19 may have no symptoms at all. Serious outcomes of COVID-19 can include hospitalization or even death. Older adults and people of any age with underlying medical conditions have a higher risk of severe illness from COVID-19. A full list of symptoms of COVID-19 can be found here: https://www.cdc.gov/coronavirus/2019-ncov/symptoms-testing/symptoms.html COVID-19 is contagious and can be spread even before a person shows symptoms of being sick (e.g. fever, coughing, difficulty breathing). Some people may test positive for COVID-19, but not have symptoms of infection. These people are considered asymptomatic but may still be able to transmit infection to others. Studies have suggested that asymptomatic infection may be common.

What are common symptoms of COVID-19?
Symptoms may appear 2-14 days after exposure and may include fever, cough, shortness of breath, fatigue, muscle or body aches, headaches, loss of sense of taste or smell, sore throat, congestion or a runny nose, nausea or vomiting and diarrhea. It is possible for an infected person to experience no symptoms at all.

How does the virus spread?
The virus that causes COVID-19 is thought to spread mainly from person to person, mainly through respiratory droplets produced when an infected person coughs or sneezes. These droplets can land in the mouths or noses of people who are nearby or possibly be inhaled into the lungs. These respiratory droplets contain virus particles which can also survive on surfaces for several hours. This is another important source of spread with COVID-19 when people touch these infected surfaces and then touch their faces (mouth, nose, eyes). Spread is more likely when people are in close contact with one another (within about 6 feet). COVID-19 seems to be spreading easily and sustainably in the community ("community spread") in many affected geographic areas. Community spread means people have been infected with the virus in an area, including some who are not sure how or where they became infected.

I tested positive for COVID-19. When can I be with others again? If you have no symptoms at time of testing and continue to have no symptoms:
• After self-isolating for 10 days since your positive test result.

If you have symptoms at time of testing or develop symptoms:
- After self-isolating for at least 10 days since symptoms first appeared and;
- At least 24 hours with no fever (without fever-reducing medications) and;
- Other symptoms of COVID-19 have improved (excluding loss of taste and smell, which may persist for weeks or months after recovery) For the most current information on CDC recommendations regarding self-isolation, visit (https://www.cdc.gov/coronavirus/2019-ncov/if-you-are sick/isolation.html)

What can I do to stay healthy during the COVID-19 pandemic?
To protect your friends, family, community, and yourself, follow these hygiene practices to help stop the spread of infections.
- Clean and wash your hands often with soap and water or an alcohol-based hand sanitizer.
- Clean all frequently touched surfaces daily with household disinfectants.
- Wear a face covering if you must be around other people in public places, in close contact with people outside of your household or where social distancing of 6 ft is difficult to maintain.
- Sneeze or cough into your elbow or into a tissue. Discard the tissue after using and wash your hands.
- Avoid close contact with people who are sick. This is especially important if you are in the high-risk group.
- If you become sick, avoid other household members where possible isolate yourself in your own room and avoid sharing bathrooms and personal items such as cups, plates and cutlery.

When should I seek medical attention?
If you develop any of the emergency warning signs (refer to User Safety section) for COVID-19 you must seek medical attention.
Emergency warning signs include*:
- Trouble breathing
- Persistent chest pain
- New confusion or inability to wake up or stay awake
- Bluish lips or face

In addition, if you are in the high-risk group (refer to User Safety section) or your symptoms are persisting or worsening, or you have concerns you should seek medical attention.
* This list is not all inclusive. Please see your healthcare professional for any other symptoms that are severe or concerning. For up-to-date information on COVID-19 please visit the CDC COVID-19 website: https://www.cdc.gov/coronavirus/2019-ncov/index.html

DISPOSAL
Dispose of all samples and materials used to perform the test in general waste. Laboratory chemical and biohazard wastes must be handled and discarded in accordance with all local, state, and national regulations.

FR REF Q-NCOV-03G

STANDARD Q
COVID-19 Ag Home Test

STANDARD™ Q COVID-19 Ag Home Test

Guide vidéo

TEST AUTO-DIAGNOSTIC
Résultats rapides en 15 minutes | Test nasal

Test antigénique COVID-19
Mode d'emploi

SD BIOSENSOR

Nom du produit : STANDARD™ Q COVID-19 Ag Home Test
Nom du modèle : STANDARD™ Q COVID-19 Ag Home Test (Q-NCOV-03G)
Utilisation prévue : STANDARD Q COVID-19 Ag Home Test est un test par immunochromatographie qui permet d'identifier de manière qualitative les antigènes nucléocapsides du SARS-CoV-2 présents dans l'échantillon nasal humain. Il fournit seulement un résultat de test de dépistage initial. Le résultat de ce test ne doit pas être utilisé comme seul et unique critère de diagnostic. D'autres tests de confirmation sont requis. Pour le programme de tests en série, si le résultat est «négatif», des tests de confirmation supplémentaires avec un test moléculaire pour les résultats négatifs peuvent être nécessaires. Cela signifie qu'il existe une forte probabilité de COVID-19, comme une personne en contact étroit avec le COVID-19 ou avec une exposition suspectée au COVID-19 ou dans des communautés à forte prévalence d'infection. Si le résultat est «positif», des tests de confirmation supplémentaires avec un test moléculaire pour des résultats positifs peuvent être nécessaires. Cela signifie qu'il existe une faible probabilité de COVID-19, par exemple chez un individu sans exposition connue au COVID-19 ou résidant dans des communautés à faible prévalence d'infection.

[Quand utiliser ce kit]
Utilisez-le
- Si vous voulez diagnostiquer une infection à COVID-19 en cours.
- Si vous êtes inquiet d'avoir contracté COVID-19.

Ne l'utilisez pas
- Si vous ne pouvez pas prélever l'échantillon conformément aux recommandations.
- Si vous êtes sujet à des saignements de nez.

EXPLICATION ET RÉSUMÉ
■ Introduction
Le Coronavirus est un virus à ARN simple brin de polarité positive avec une enveloppe de 80 à 120 nm de diamètre. Son matériel génétique est le plus grand de tous les virus à ARN et est un pathogène important des maladies chez de nombreux animaux domestiques, de compagnie et chez les humains. Il peut provoquer de nombreuses maladies aiguës ou chroniques. Les symptômes indiquant qu'une personne est infectée par le coronavirus sont les suivants : problèmes respiratoires, fièvre, toux, souffle court et dyspnée. Dans les cas les plus graves, l'infection peut entraîner une pneumonie, le syndrome respiratoire aigu sévère, des défaillances rénales et même la mort. Le « 2019-nCov » a été découvert à Wuhan en 2019 par des cas de pneumonies virales. L'Organisation mondiale de la santé l'a nommé ainsi le 12 janvier 2020, confirmant qu'il peut provoquer des maladies comme le Syndrome Respiratoire du Moyen-Orient (MERS) et le syndrome respiratoire aigu sévère (SARS).

■ Principe du test
STANDARD Q COVID-19 Ag Home Test comprend deux lignes recouvertes, "C" (ligne de contrôle) et "T" (ligne de test) sur la surface de la membrane en nitrocellulose. Ces deux lignes (contrôle et test) situées dans la fenêtre de résultats ne sont pas visibles avant d'avoir introduit des échantillons. Des anticorps anti-SARS-CoV-2 monoclonaux de souris recouvrent la zone de la ligne de test et des anticorps IgY anti-poulet monoclonaux de souris recouvrent la zone de la ligne de contrôle. Les anticorps anti-SARS-CoV-2 monoclonaux de souris conjugués aux particules de couleur sont utilisés comme des détecteurs pour le dispositif antigène SARS-CoV-2. Lors du test, les antigènes SARS-CoV-2 dans l'échantillon interagissent avec les anticorps anti-SARS-CoV-2 monoclonaux conjugués aux particules de couleur pour former un complexe de particules de couleur anticorps-antigènes. Ce complexe migre sur la membrane par action capillaire jusqu'à la ligne de test où il sera capturé par les anticorps anti-SARS-CoV-2 monoclonaux de souris. Une ligne de test colorée devrait apparaître dans la fenêtre de résultats si les antigènes SARS-CoV-2 sont présents dans l'échantillon. L'intensité de la ligne de test colorée variera en fonction de la quantité d'antigènes SARS-CoV-2 présents dans l'échantillon. Si aucun antigène SARS-CoV-2 n'est présent dans l'échantillon, aucune couleur n'apparaîtra sur la ligne de test. La ligne de contrôle est utilisée en guise de contrôle procédural et doit toujours apparaître si la procédure de test est lancée correctement et que les réactifs de test de la ligne de contrôle sont valides.

CONTENUS
Dispositif de test, Tube de solution & capuchon de buse, Écouvillon stérile, Mode d'emploi & Guide de référence rapide

MODE D'EMPLOI
■ Prélèvement de l'échantillon
[Écouvillon nasal]
1. Ouvrez le sceau du tube de solution et placez le tube de solution sur le trou du support de l'emballage.
2. Ouvrez le sachet d'écouvillon stérile.
3. Inclinez la tête en arrière de 70 degrés.
4. Tout en tournant l'écouvillon, insérez-le à moins d'un pouce (environ 1,5 cm).
5. Tourner l'écouvillon 10 fois contre les parois nasales.
6. Répéter l'opération dans l'autre narine, en utilisant le même écouvillon.

■ Préparation
1. Lire attentivement le mode d'emploi pour utiliser STANDARD Q COVID-19 Ag Home Test.
2. Vérifier la date de péremption à l'arrière du sachet en aluminium. Il ne faut pas utiliser le kit, si la date de péremption est dépassée.
3. Vérifier le dispositif de test et le paquet avec le desiccant dans le sachet en aluminium.

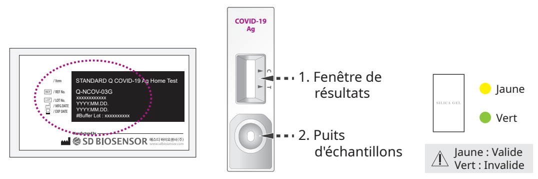

■ Procédure du test
1. Insérer l'écouvillon dans un tube de solution. Remuer le tube de solution plus de 10 fois, tout en pressant sur le tube.
2. Retirer l'écouvillon tout en appuyant sur les côtés du tube pour en extraire le liquide.
3. Visser fermement le capuchon de buse sur le tube.
4. Verser 4 gouttes de l'échantillon mélangé au puits d'échantillons du dispositif de test.
5. Lire le résultat du test au bout de 15 minutes.

⚠ Ne pas lire les résultats du test après 30 minutes. Les résultats pourraient être erronés.

INTERPRÉTATION DES RÉSULTATS DE TEST

Résultats	Exemple	Interprétation des résultats
Négatif	C T	Résultat négatif : une bande colorée apparaîtra uniquement sur la ligne de contrôle(C) de la fenêtre de résultats.
Positif	C T / C T	Résultat positif : des bandes colorées apparaîtront sur la ligne de contrôle(C) et sur la ligne de test(T) de la fenêtre de résultats.
Relancer le test	C T / C T	Relancer le test : si la ligne de contrôle(C) n'apparaît pas dans la fenêtre de résultats, le résultat est invalide. Relancer le test en utilisant un nouveau échantillon et un nouveau dispositif.

* Les résultats positifs doivent être pris en compte par rapport à l'historique clinique et d'autres données disponibles.
* Même si la ligne de contrôle est faible, ou si la ligne de test n'est pas uniforme, le test devra être considéré comme fonctionnant correctement et le résultat du test devra être interprété comme un résultat positif.

KIT DE STOCKAGE ET STABILITÉ
Stocker le kit à une température entre 2 et 30 °C (36 - 86 °F), à l'abri de la lumière directe du soleil. Les contenus du kit peuvent être utilisés et restent stables jusqu'à la date d'expiration indiquée à l'extérieur de la boîte. Ne pas stocker le kit congelé.

PRÉCAUTIONS D'EMPLOI
1. Ce produit doit être utilisé pour un diagnostic in vitro.
2. Le test d'autodiagnostic COVID-19 est un nouveau dispositif médical de test antigénique au coronavirus qui utilise un échantillon nasal.
3. Étant donné que le test rapide antigénique SARS-CoV-2 est un test de dépistage, il est recommandé d'effectuer régulièrement ce test.
4. Avant d'utiliser ce test, il est important de lire le mode d'emploi et de suivre la procédure de test.
5. Ce produit a été évalué cliniquement sur des échantillons de patients après l'apparition des premiers symptômes du COVID-19, et n'a pas été évalué cliniquement sur des patients asymptomatiques.
6. Après le test, il faut se laver les mains rigoureusement avec du savon et de l'eau courante.

■ Précautions concernant l'analyse des résultats
1. Il n'est pas possible de diagnostiquer précisément une infection par le SARS-CoV-2 en s'appuyant uniquement sur le résultat de ce produit. Lorsqu'il est soumis à un examen approfondi, le docteur doit prendre la décision finale en tenant en compte les symptômes cliniques après avoir effectué un test de confirmation avec un produit RT-PCT approuvé (sauf pour les dépistages d'urgence).
2. Si la concentration d'antigènes du SARS-CoV-2 dans l'échantillon est inférieur à la limite de détection du test, ou si l'échantillon est mal prélevé ou mal transporté, le résultat peut être un faux-négatif. Par conséquent, la possibilité d'une infection par le SARS-CoV-2 ne peut pas être écartée lorsque le résultat est négatif.
3. Ce produit vérifie uniquement la présence des antigènes du SARS-CoV-2, et il n'existe aucune corrélation entre la force de la ligne de test et la concentration d'antigènes du SARS-CoV-2.
4. Si une mutation apparaît dans la zone de liaison des anticorps monoclonaux contenus dans ce produit, la sensibilité peut être réduite.
5. Ce produit ne peut pas distinguer les antigènes du SARS-CoV et du SARS-CoV-2.
6. Ce produit vérifie uniquement la présence des antigènes du SARS-CoV-2, et il n'existe aucune corrélation entre la force (ou la valeur mesurée) de la ligne de test et la concentration d'antigènes du SARS-CoV-2.
7. Les échantillons prélevés après 6 jours à partir de l'apparition des symptômes peuvent donner des résultats faux-négatifs.

APRÈS LE TEST
Après le test, veuillez suivre les instructions ci-dessous.

■ Résultat positif à la COVID-19.
Si le résultat est positif, Il est très probable que vous ayez le COVID-19.

[Ce qu'il faut faire]
1. Vous devez vous isoler pour éviter de transmettre le virus aux autres. Il existe une toute petite possibilité pour que le test donne un résultat positif qui est faux (un résultat faux-positif).
2. Consulter un professionnel de santé dès que possible. Votre médecin déterminera avec vous le meilleur traitement en fonction des résultats du test mais également en prenant en compte d'autres facteurs, notamment vos antécédents médicaux, vos symptômes, votre exposition possible et les endroits où vous avez récemment été.

■ Résultat négatif à la COVID-19.
Un résultat de test négatif signifie que les antigènes du SARS-CoV-2 qui causent COVID-19 n'ont pas été trouvés dans votre échantillon. Pour la COVID-19, un résultat de test négatif pour un échantillon prélevé alors qu'une personne a des symptômes signifie généralement que ce n'est pas la COVID-19 qui a causé la maladie actuelle.

[Ce qu'il faut faire]
Veuillez consulter un professionnel de santé si vous développez des symptômes, si les symptômes persistent ou s'ils s'aggravent. Il est possible que le test fournisse un résultat négatif qui est incorrecte (un faux-négatif) chez des personnes infectées par la COVID-19. Cela signifie que vous pouvez avoir contracté COVID-19 même si le test est négatif. Si c'est le cas, votre médecin traitant prendra en considération le résultat du test avec tous les autres éléments associés à vos antécédents médicaux (comme des symptômes, la durée de la maladie, les expositions possibles, et les endroits où vous avez récemment été) pour savoir comment vous prescrire le bon traitement. Il est important que vous collaboreriez avec votre médecin pour comprendre les étapes qu'il faut suivre par la suite.

■ Résultat invalide
Un résultat de test invalide signifie que le test a rencontré une erreur (voir ci-dessous).

[Les raisons qui entraînent une erreur]
- Lorsque le test est effectué sans une réelle compréhension de la façon dont il doit être mené
- Lorsque l'échantillon utilisé est insuffisant ou excessif
- Lorsque l'utilisateur vérifie le résultat en dehors de la durée de lecture requise

[Ce qu'il faut faire]
Lorsque le test a rencontré une erreur, vous devez relancer l'opération avec un nouveau test ou consulter un professionnel de santé. Avant d'obtenir le résultat du nouveau test, vous devez vous isoler chez vous. Si vous avez des questions, n'hésitez pas à nous contacter via l'assistance téléphonique 1-888-885-6121 /, par email help.covid19@sdbiosensor.com ou via notre site Internet www.sdbiosensor.com.

SÉCURITÉ DE L'UTILISATEUR
[Ce qu'il faut savoir]
Les adultes et personnes plus âgées qui souffrent de problèmes médicaux sous-jacents comme des maladies cardiaques ou pulmonaires ou du diabète semblent être plus à risque de développer des formes plus graves de la COVID-19.

[Groupes à haut risque]
- Les personnes âgées de 65 ans et plus
- Les personnes dans les maisons de repos ou dans des établissements de soins de longue durée
- Les personnes de tout âge souffrant des problèmes de santé sous-jacents suivants, surtout s'ils ne sont pas bien contrôlés :
  - Cancer
  - Maladie pulmonaire obstructive chronique
  - Insuffisance rénale chronique
  - Immunodépression comme après une transplantation d'un organe solide
  - Obésité (IMC >30)
  - Problèmes cardiaques graves comme une crise cardiaque, une coronaropathie, des cardiomyopathies
  - Drépanocytose
  - Diabètes de Type II
  - Immunodépression
  - Asthme (Modéré-sévère)
  - Maladie neuro-vasculaire (affectant les vaisseaux sanguins jusqu'au cerveau)
  - Mucoviscidose
  - Hypertension artérielle
  - Immunodépression (système immunitaire affaibli) transfusion sanguine ou transplantation de moelle osseuse, immunodéficiences, VIH, utilisation de corticostéroïdes, utilisation d'autres médicaments affaiblissant le système immunitaire
  - Pathologies neurologiques comme la démence
  - Maladie hépatique
  - Grossesse
  - Fibrose pulmonaire
  - Fumer
  - Thalassémie
  - Diabètes de Type I

[Quand appeler les urgences]
Reconnaître les signaux d'alerte* graves de la COVID-19. Si une personne présente l'un des symptômes suivants, appeler immédiatement les urgences :
- Difficultés à respirer
- Douleur persistante ou pression sur la poitrine
- Confusion nouvelle
- Impossibilité de se réveiller ou de rester éveillé
- Lèvres ou visage bleuâtres

*Cette liste n'est pas une liste exhaustive des symptômes. Veuillez appeler votre médecin traitant si vous souffrez de tout autre symptôme grave ou inquiétant.

Appeler le 911 ou appeler à l'avance votre centre d'urgence local :
Indiquer à l'opérateur que vous souhaitez obtenir de l'aide pour quelqu'un qui a contracté ou qui a peut-être contracté la COVID-19.

FAQ
Quels sont les risques et bénéfices potentiels de ce test ?

Les risques potentiels sont les suivants:
- Inconfort possible lors du prélèvement de l'échantillon.
- Résultats de test incorrects possibles.
  - Les bénéfices potentiels sont les suivants : Les résultats, associés à d'autres informations, peuvent aider les professionnels de santé informés à prescrire le bon traitement.
  - Les résultats de ce test peuvent aider à limiter le risque de transmettre la COVID-19 à votre famille ou à d'autres personnes de votre entourage.
  - Vous avez le choix de refuser ce test. Cependant, votre médecin vous a prescrit ce test car il pense que les résultats pourront l'aider à proposer le bon traitement.

PLUS D'INFORMATIONS CONCERNANT CE TEST
1. Ce produit contient de petites quantités de matières d'origine animale.
2. Si vous avez des questions concernant ce produit, veuillez contacter votre distributeur local.
3. Vous pouvez également consulter le site Internet www.sdbiosensor.com pour visionner des démonstrations produits.

INFORMATION CONCERNANT LA COVID-19
Qu'est-ce que COVID-19 ?
COVID-19 est la maladie causée par le virus SARS-CoV-2, un nouveau virus chez l'humain entraînant des maladies respiratoires contagieuses. COVID-19 peut se manifester à travers des symptômes modérés à graves même si certaines personnes infectées peuvent ne présenter aucun symptôme. Les conséquences graves de la maladie peuvent être une hospitalisation ou même la mort. Les adultes et personnes plus âgées qui souffrent de problèmes médicaux sous-jacents présentent un risque plus élevé de développer des formes plus graves de la COVID-19. Une liste complète des symptômes de la COVID-19 est accessible ici : https://www.cdc.gov/coronavirus/2019-ncov/symptoms-testing/symptoms.html. COVID-19 est une maladie contagieuse et peut se transmettre avant même qu'une personne ne montre des symptômes de la maladie (ex : fièvre, toux, difficultés à respirer). Certaines personnes peuvent avoir un test positif à COVID-19, mais ne présenter aucun des symptômes de l'infection. Ces personnes sont considérées comme asymptomatiques mais peuvent toujours transmettre le virus aux autres. Les études suggèrent que l'infection asymptomatique est peut-être commune.

Quels sont les symptômes communs de la COVID-19 ?
Les symptômes peuvent apparaitre entre 2 et 14 jours après exposition au virus. Les symptômes sont les suivants : fièvre, toux, souffle court, fatigue, douleurs musculaires et corporelles, céphalées, perte du goût et de l'odorat, mal de gorge, congestion ou écoulement nasal, nausées ou vomissements et diarrhée. Il est possible qu'une personne infectée ne présente aucun symptôme du tout.

Comment le virus se transmet-il ?
On considère que la transmission s'effectue essentiellement de personne à personne par l'exposition à des gouttelettes, provenant de la personne infectée, expulsées lors d'une toux ou d'éternuements. Ces gouttelettes peuvent atterrir dans la bouche et le nez des personnes aux alentours et être inhalées jusque dans les poumons. Ces gouttelettes expulsées contiennent des particules de virus qui sont également capables de survivre sur des surfaces pendant plusieurs heures. Il existe une autre source importante de transmission de la maladie : lorsque des personnes touchent des surfaces infectées puis portent les mains à leur visage (bouche, nez, yeux). La transmission est plus probable lorsque des personnes sont en contact rapproché (moins de 2 m). La COVID-19 semble se propager facilement et durablement au sein d'une communauté (« propagation au sein d'une communauté ») dans de nombreuses zones géographiques touchées. La propagation au sein d'une communauté signifie des personnes infectées par le virus dans une région, notamment celles qui ne sont pas sûres de savoir comment et où elles ont été infectées.

Je suis positif à la COVID-19. Quand pourrais-je à nouveau entrer en contact avec les autres ? Si vous n'avez aucun symptôme au moment du test et que vous continuez à ne ressentir aucun symptôme :
• Après vous être isolé pendant 10 jours après votre résultat positif.

Si vous avez des symptômes au moment du test ou développez des symptômes :
- Après vous être isolé pendant 10 jours à partir du début de l'apparition des premiers symptômes et ;
- Au moins 24 heures sans fièvre (sans avoir pris de médicaments qui atténuent la fièvre) et ;
- Que les autres symptômes se sont améliorés (sauf la perte de goût et d'odorat qui eux, peuvent persister des semaines ou des mois après la guérison). Si vous souhaitez en savoir plus sur les recommandations du CDC concernant l'isolation, consulter (https://www.cdc.gov/coronavirus/2019- ncov/if-you-are sick/isolation.html)

Que puis-je faire pour rester en bonne santé pendant la pandémie de COVID-19 ?
Pour protéger vos amis, votre famille, votre entourage, et vous-même, respecter les mesures d'hygiène suivantes afin de casser la chaîne de contamination.
- Nettoyer et lavez-vous les mains souvent avec du savon et de l'eau ou un gel hydro-alcoolique.
- Nettoyer quotidiennement toutes les surfaces les plus fréquemment touchées avec des désinfectants ménagers.
- Porter un masque si vous devez être en contact avec d'autres personnes dans des espaces publics, en contact rapproché avec d'autres personnes à l'extérieur de votre domicile ou là où la distanciation sociale de 2 mètres est difficile à appliquer.
- Éternuer ou tousser dans votre coude ou dans un mouchoir en papier. Jeter le mouchoir en papier après l'avoir utilisé et lavez-vous les mains.
- Éviter tout contact rapproché avec des personnes qui sont malades. C'est d'autant plus important lorsque vous faites partie des personnes à risque.
- Si vous tombez malade, évitez les autres membres de votre foyer dès que c'est possible, isolez-vous dans votre chambre et évitez de partager tout article de toilette et objet personnel comme des verres, des assiettes et des couverts.

Quand dois-je consulter un médecin?
Si vous développez le moindre signal d'alerte (voir la partie Sécurité de l'utilisateur), vous devez contacter un médecin. Les signaux d'alerte graves sont les suivants* :
- Difficultés à respirer
- Douleur dans la poitrine persistante
- Confusion nouvelle ou incapacité à se réveiller ou à rester éveillé
- Lèvres et visage bleuâtres

Par ailleurs, si vous faites partie des personnes à haut risque (voir la partie Sécurité de l'utilisateur), si vos symptômes persistent ou s'aggravent, ou si vous avez des inquiétudes, n'hésitez pas à contacter votre médecin traitant.
* Cette liste est non exhaustive. Veuillez contacter un professionnel de santé si vous présentez d'autres symptômes graves ou inquiétants. Pour rester à jour sur la pandémie de COVID-19, consulter le site Internet du CDC : https://www. cdc. gov/coronavirus/2019-ncov/index.html

DISPOSITION
Éliminer tous les échantillons et matériaux utilisés pour effectuer le test dans les déchets ordinaires. Les déchets chimiques et biologiques de laboratoire doivent être manipulés et jetés conformément à toutes les réglementations locales, régionales et nationales.

SPECIFIC PERFORMANCE DATA
■ Clinical evaluation
The clinical performance of the STANDARD Q COVID-19 Ag Home Test for patient self-testing was evaluated using nasal swab samples collected from 146 (of which, 139 within 7 days post symptom onset) study participants in a prospective study at a clinical center in Germany. The clinical evaluations were performed independently from the manufacturer and distributor within a collaboration between the university hospitals Charité in Berlin and Heidelberg. The study cohort included symptomatic adults (aged 18 to 68) who were clinically suspected of having a SARS-CoV-2 infection. In the patient self-testing group, the study participants followed written instructions with illustrations for taking a nasal swab sample and performing the test themselves. The samples were collected and the tests performed under the observation of healthcare professionals, who did not intervene at any stage. PCR tests using combined deep nose/deep throat swab samples were used as a comparative method. Nasal sampling by the self-testers always preceded the combined deep nose/deep throat sample collection for RT-PCR comparison. A SARS-CoV-2 infection was diagnosed (using PCR) in 27.4 % of the patients. The clinical performance of the STANDARD Q COVID-19 Ag Home Test was also evaluated for professional testing following patient self-collection and professional collection of nasal swab samples in the same clinical center. 229 adults who were clinically suspected of having a SARS-CoV-2 infection were included in the prospective study. 133 study participants (there of 126 within 7 days post symptom onset) underwent nasal sampling performed by healthcare professionals and 96 study participants (thereof 83 within 7 days post symptom onset) followed instructions for collecting their nasal swab samples themselves. Self-collection was performed under the supervision of healthcare professionals. PCR tests were performed as described above.

TEST SENSITIVITY AND SPECIFICITY
In the self-testing study, the STANDARD Q COVID-19 Ag Home Test correctly identified 91.2 %(CI: 76.3 % - 98.1 %) of infected study participants with a relatively high viral load (Ct ≤ 30). Individuals with a high viral load are considered to be at higher risk of being infectious and transmitting the virus to others. For all study participants, the antigen rapid test correctly identified 82.5 % (CI: 67.2 % - 92.7 %) of infected study participants and 100.0 % (CI: 96.5 % - 100.0 %) of non-infected study participants. In all 3 cohorts together, 110 PCR-positive and 263 PCR-negative study participants were evaluated using the STANDARD Q COVID-19 Ag Home Test. For patients with a relatively high viral load (Ct ≤ 30), the relative sensitivity was 91.1 % (95 % CI: 83.8 % - 95.8 %, N=101). For all samples, the overall relative sensitivity and the overall relative specificity were 86.4 %(95 % CI: 78.5 % - 92.2 %) and 99.6 % (95 % CI: 97.9 % - 100.0 %), respectively. For patients tested within 7 days post symptom onset (DPSO), the relative sensitivity was 87.4 % (95 % CI: 79.4 % - 93.1 %) and the relative specificity was 99.6 % (95 % CI: 97.7 % - 100.0 %).

	Antigen positive/ PCR positive	Antigen negative/ PCR negative	Relative sensitivity (95% confidence interval)	Relative specificity (95% confidence interval)
Self Testing**	33 out of 40	105 out of 105	82.5 % (67.2 % - 92.7 %)	100 % (96.5 % -100 %)
Self collection	31 out of 34	61 out of 62	91.2 % (76.3 % - 98.1 %)	98.4 % (91.3 % - 100 %)
Professional collection*	31 out of 36	96 out of 96	86.1 % (70.5 % - 95.3 %)	100 % (96.2 % - 100 %)
Combined*,**	95 out of 110	262 out of 263	86.4 % (78.5 % - 92.2 %)	99.6 % (97.9 % - 100 %)
Ct ≤ 30***	92 out of 101	n.a.	91.1 % (83.8 % - 95.8 %)	n.a.
DPSO ≤ 7*,**	90 out of 103	242 out of 243	87.4 % (79.4 % - 93.1 %)	99.6 % (97.7 % - 100 %)

*One sample was excluded from the analysis because the PCR test result was not available.
**One sample (PCR negative) was excluded from the analysis because the antigen test result was not available.
***Ct values are commonly used to estimate the amount of the viral material in samples. A low Ct value suggests the presence of a lot of viral material, and a high Ct value suggests the presence of lower levels of viral material.

BIBLIOGRAPHY

1. Clinical management of severe acute respiratory infection when novel coronavirus(nCoV) infection is suspected. Interim guidance. WHO.2020
2. Diagnostic detection of Wuhan coronavirus 2019 by real-time RT-PCR.2020
3. Diagnosis and treatment of pneumonia caused by new coronavirus (trial version 4) National Health Commission. 2020

SYMBOL

Symbol	Meaning	Symbol	Meaning	Symbol	Meaning	Symbol	Meaning	Symbol	Meaning	Symbol	Meaning
REF	Reference number		Caution		Use by	LOT	Batch code		Consult Instructions for Use		Do not re-use
IVD	*In vitro* Diagnostics		Note		Manufacturer		Date of manufacture	Σ	Contains Sufficient for <n> Tests		Keep away from sunlight
	Indicate that you should keep the product dry		To indicate the temperature limitations in which the transport package has to be kept and handled.				Do not use if packaging is damaged				

IVD

Manufactured by SD Biosensor, Inc.
Head office : C-4th&5th, 16, Deogyeong-daero 1556beon-gil, Yeongtong-gu, Suwon-si, Gyeonggi-do, 16690, REPUBLIC OF KOREA
Manufacturing site : 74, Osongsaengmyeong 4-ro, Osong-eup, Heungdeok-gu, Cheongju-si, Chungcheongbuk-do, 28161, REPUBLIC OF KOREA

For inquiries, please contact us at help.covid19@sdbiosensor.com

L20COV13HML14R1
Issue date:2021.06

STANDARD Q

COVID-19 Ag Home Test

EN

COVID-19 Antigen Home Test Quick reference instruction

Fast result in 15 minutes | Nasal Test

SD BIOSENSOR

!

Study the Instructions for Use and User Manual thoroughly before using Quick Reference Instructions. This is not a complete Instructions for Use.

After looking at the diagram below and familiarizing yourself with how to use it, follow the instructions below.

Video Guide

STEP 1 PREPARATION

1 Wash your hands.

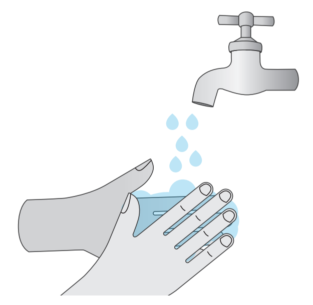

! It is recommended to wear gloves when using the product.

2 Check the kit contents before testing.

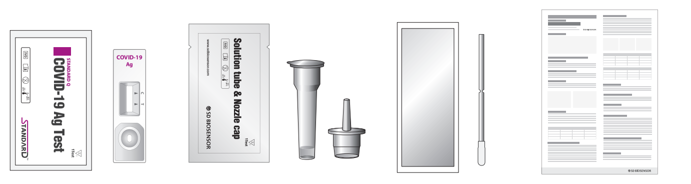

3 Check the expiry date at the back of the foil pouch. After open the foil pouch, check the test device and the desiccant pack in the foil pouch.

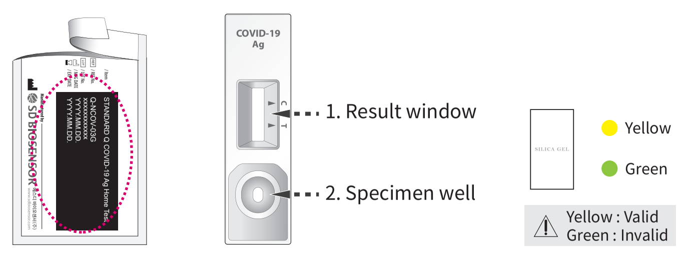

STEP 2 SAMPLE COLLECTION

4 Open the Solution tube & Nozzle cap pouch and the seal of solution tube.

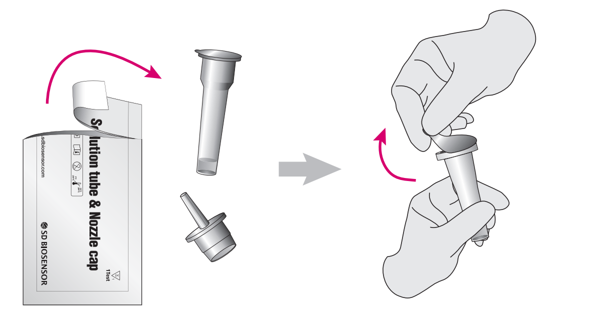

5 Set the solution tube on the stand hole of the package.

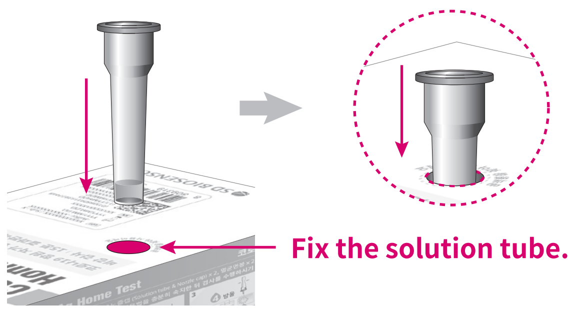

6 Open the sterile swab pouch and hold the swab.

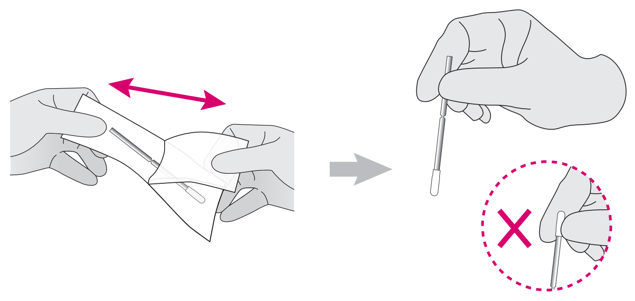

! Do not touch swab tip when handling swab sample.

7 Insert the sterile swab and rotate for both side of nostrils. The sterile swab should be entered in less than one inch (about 1.5cm).

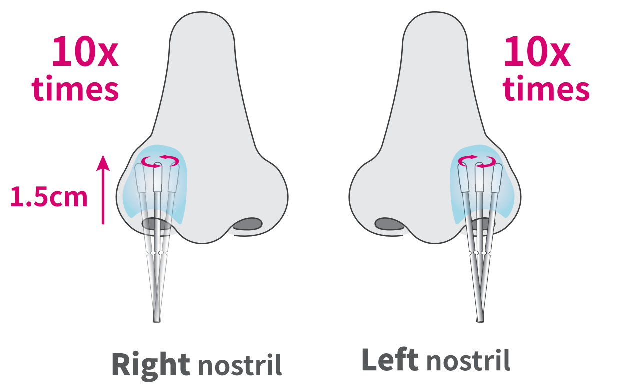

! Do not soak the swab in the solution tube or other liquid until you put it into the nasal cavity.

STEP 3 TEST PROCEDURE

8 Insert the swab into an solution tube. While squeezing the solution tube, stir the swab more than 10 times. Remove the swab while squeezing the sides of the tube to extract the liquid from the swab.

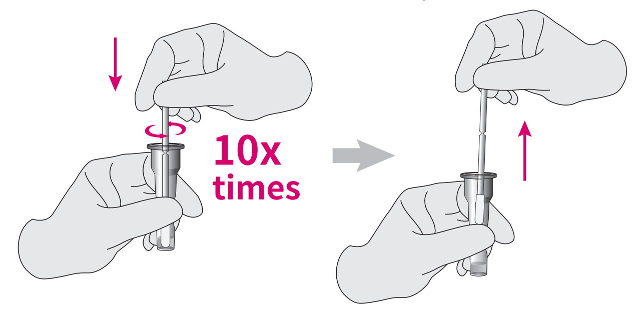

! In case of contact with your skin or eyes, wash immediately with plenty of water.

9 Press the nozzle cap tightly onto the tube.

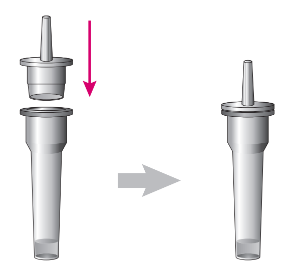

10 Apply 4 drops of extracted sample to the sample well of the test device.

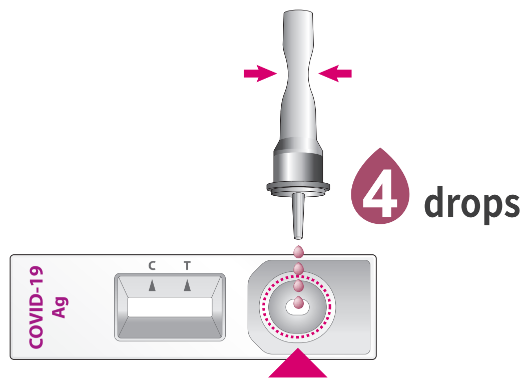

! Do not squeeze out all of the solution in the solution bottle.

11 Read the test result in 15 minutes.

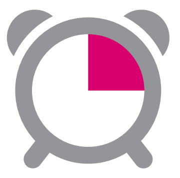

! Do not read test results after 30 minutes.

STEP 4 INTERPRETATION OF TEST RESULT

Negative

Negative result: A colored band will appear only control line(C) on the result window.

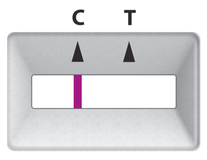

Positive

Positive result: Colored bands will appear both control line(C) and test line(T) on the result window.

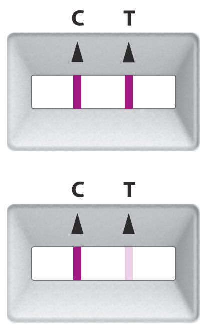

Invaild

Retest: If the control line(C) is not appeared in the result window, it is an invalid result. Retest using a new sample and device.

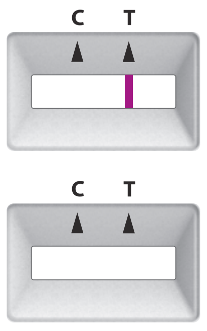

* Positive results should be considered in conjunction with the clinical history and other data available.

* Even if the control line is faint, or the test line isn't uniform, the test should be considered to be performed properly and the test result should be interpreted as a positive result.

For inquiries, please contact us at help.covid19@sdbiosensor.com

Head office: C-4&5Floor, 16, Deogyeong-daero 1556beon-gil, Yeongtong-gu, Suwon-si, Gyeonggi-do, 16690, REPUBLIC OF KOREA
© 2021 SD BIOSENSOR. All rights reserved.

STANDARD Q

COVID-19 Ag Home Test

FR

Autotest antigénique Covid-19 Guide de démarrage rapide

Résultat rapide en 15 minutes | Test nasal

SD BIOSENSOR

!

Lire attentivement le mode d'emploi avant d'utiliser le guide de démarrage rapide. Il ne s'agit pas d'un mode d'emploi complet.

Après avoir regardé la vidéo pour vous familiariser avec l'utilisation du test, veuillez suivre les instructions ci-dessous.

Vidéo d'aide

ÉTAPE 1 PRÉPARATION

1 Lavez-vous les mains.

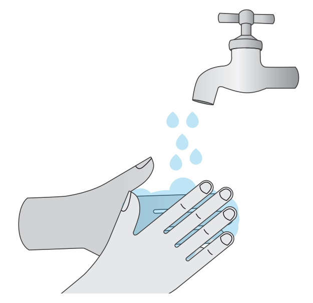

! Il est recommandé de porter des gants lorsque vous utilisez le produit.

2 Contrôlez les contenus du kit avant de procéder au test.

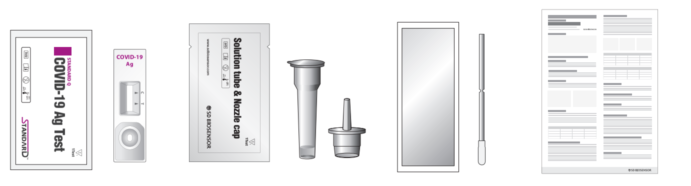

3 Vérifier la date de péremption à l'arrière de la pochette aluminium. Après avoir ouvert la pochette aluminium, contrôlez la cassette test et le sachet de dessicant qu'elle contient.

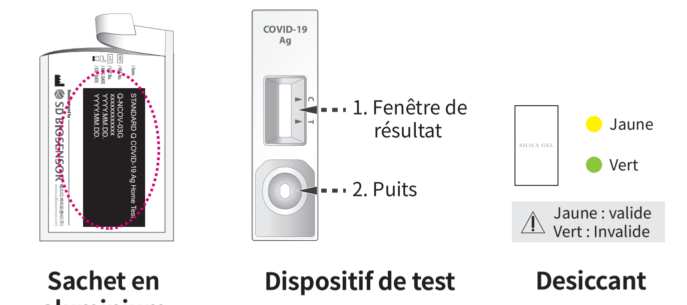

ÉTAPE 2 PRÉLÈVEMENT DE L'ÉCHANTILLON

4 Ouvrir la pochette contenant le tube de solution et la tétine compte-goutte, puis retirez l'opercule du tube de solution.

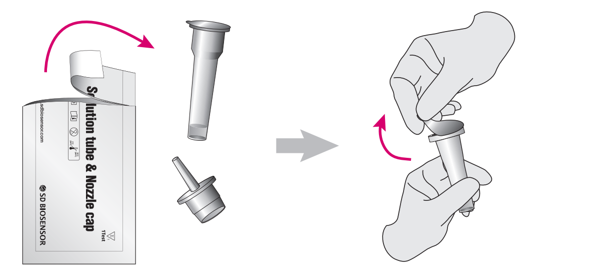

5 Placer le tube de solution dans le trou prédécoupé sur la boîte.

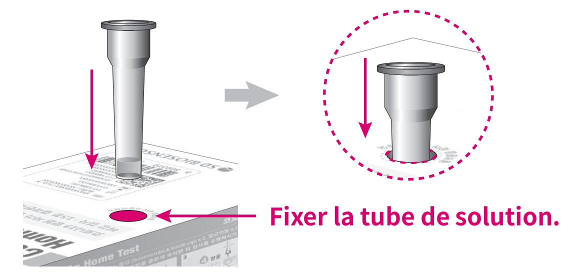

6 Ouvrir la pochette contenant l'écouvillon stérile et prendre l'écouvillon.

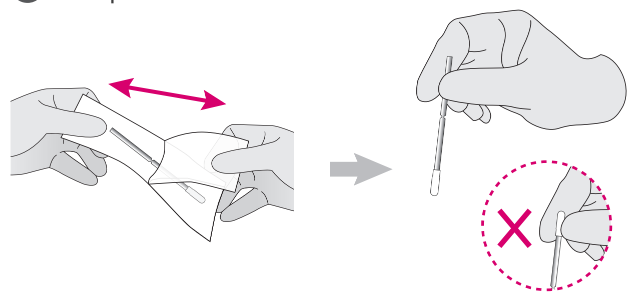

! Ne pas toucher l'extrémité de l'écouvillon lorsque vous manipulez un échantillon.

7 Inserer l'écouvillon stérile dans le nez et le faire rouler contre les parois des deux narines. Inserer l'écouvillon stérile parallèlement au palais sur moins de 1,5 cm.

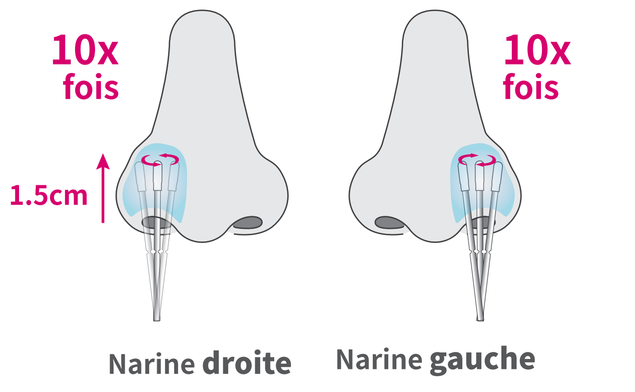

! Ne pas plonger l'écouvillon dans le tube de solution ou tout autre liquide avant de l'avoir introduit dans la cavité nasale.

ÉTAPE 3 DÉROULEMENT DU TEST

8 Inserer l'écouvillon dans le tube de solution. Tourner l'écouvillon plus de 10 fois tout en pressant le tube de solution. Retirer l'écouvillon tout en appuyant sur l'extérieur du tube afin d'extraire le liquide de l'écouvillon.

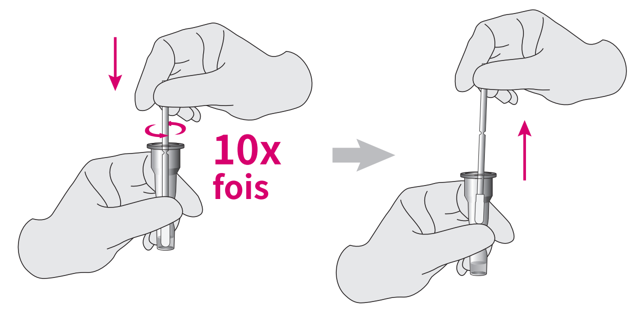

! En cas de contact avec la peau ou les yeux, rincer immédiatement et abondamment à l'eau.

9 Enfoncer correctement la tétine compte-goutte dans le tube.

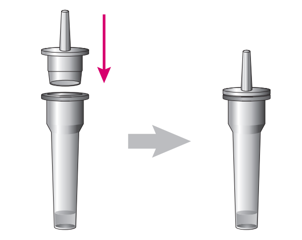

10 Déposer 4 gouttes de solution dans le puits de la cassette test.

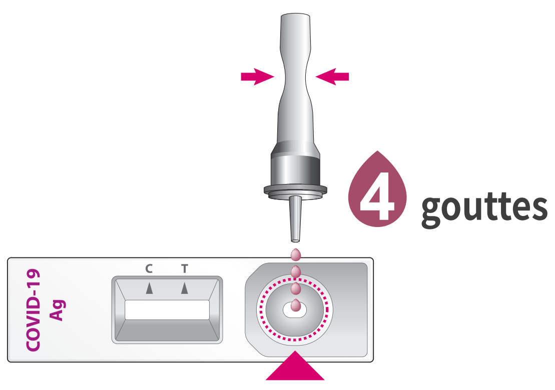

! Ne pas vider toute la solution du tube de solution.

11 Lire le résultat du test au bout de 15 minutes.

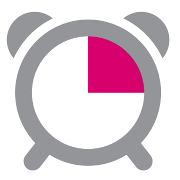

! Ne pas lire le résultat au-delà de 30 minutes.

ÉTAPE 4 INTERPRÉTATION DU RÉSULTAT DU TEST

Négatif

Résultat négatif : une bande colorée apparaît uniquement dans la zone de contrôle(C) de la fenêtre de résultat.

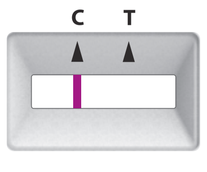

Positif

Résultat positif : des bandes colorées apparaissent dans la zone de contrôle(C) et la zone de test(T) de la fenêtre de résultat.

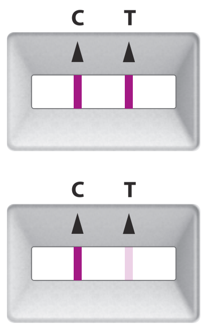

Invalide

Répéter le test : si la ligne de contrôle(C) n'apparaît pas dans la fenêtre de résultat, le résultat n'est pas valide. Répéter le test à l'aide d'une nouvelle cassette test et en prélevant un nouvel échantillon.

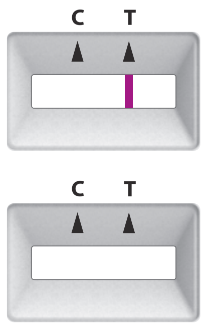

* Un résultat positif doit être pris en compte en combinaison avec les antécédents médicaux et d'autres données disponibles.

* Même si la ligne de contrôle est pâle ou la ligne de test non uniforme, le test peut être considéré comme réalisé correctement et doit être interprété comme un résultat positif.

Pour toute question contactez-nous à l'adresse help.covid19@sdbiosensor.com

Siège social : C-4&5Floor, 16, Deogyeong-daero 1556beon-gil, Yeongtong-gu, Suwon-si, Gyeonggi-do, 16690, RÉPUBLIQUE DE CORÉE
© 2021 SD BIOSENSOR. Tous droits réservés.

Q_COVID-19 Ag Home Test_IFU&QRG_L20COV13HML14R1_20210616.indd 2
2021-06-17 오전 9:17:56
