# Supplementary material for: Evaluation of COVID-19 antigen rapid diagnostic tests for self-testing in Lesotho and Zambia
Source: PLoS One. 2024 Feb 29;19(2):e0280105. doi: 10.1371/journal.pone.0280105 (PMC10903820; doi:10.1371/journal.pone.0280105)
Supplement: S2 Appendix — (PDF) [file pone.0280105.s002.pdf]

S2 Appendix. Participant Form (including Ease-of-use questionnaire) and Observation Form

Validation of self-testing with Ag-RDTs study

Participant's Form_v1.0_17012022

Participant should fill in this form after conducting the self-testing

1. Date *(dd/mm/yy)* _ _/_ _/_ _
2. My test result is: Positive ☐ Negative ☐ Invalid ☐ I don't know ☐

Only to be filled in if invalid result:

3. If Invalid, have you repeated the self-testing? Yes ☐ No ☐
4. What is the result of your second test? Positive ☐ Negative ☐ Invalid ☐ I don't know ☐

1	EASE	*How easy was the Panbio COVID AgRDT to do?*	1 = Very easy<br>2 = Somewhat easy<br>3 = Somewhat difficult<br>4 = Very difficult	☐
2	DFCTY	*Which part of the test did you find the most difficult to perform?*	**1=** Putting the buffer into the extraction tube<br>**2 =** Collecting the nasal swab.<br>**3** = Swirling the swab in the extraction tube<br>**4 =** Dispensing 5 drops from the tube into the test device<br>**5 =** Reading the test.<br>**6 =** All the steps were easy<br>7 = None of the steps were difficult<br>**7 =** Other difficulty<br>…………………………	☐
3	INSTR	*How easy was it to understand the instructions provided?*	1 = Very easy<br>2 = Somewhat easy<br>3 = Somewhat difficult<br>4 = Very difficult	☐
3.1		*If responded very difficult, explain why (open text)*	______________________________<br>______________________________<br>______________________________	
4	WRREF	*How much did you rely on the pictures to conduct the test?*	1 = Very much<br>2 = Somewhat<br>3 = A little<br>4 = Not at all	☐

5	MHELP	*Which was the most helpful piece of instruction?*	1 = Pictures<br>2 = Written instructions
6	SATIS	*Overall, how satisfied were you with the self-testing process?*	1 = Very satisfied<br>2 = Somewhat satisfied<br>3 = A little satisfied<br>4 = Not satisfied at all
6.1		*If responded Little satisfied or Not satisfied, explain why (open text)*	______________________________<br>______________________________<br>______________________________
7	RECOM	*What would you recommend to improve the experience of self-testing?*	……………………………………………<br>……………………………………………<br>……………………………………………<br>……………………………………………<br>……………………………………………
8		*Do you believe that your test <u>result</u> was <u>correct</u>?*	1 = Definitely correct<br>2 = Probably correct<br>3 = Not correct
9	RCKIT	*Would you recommend this COVID test kit for self-testing to friends and family?*	1 = Yes<br>2 = No
10	STFT	*How likely would you be to self-test in the future if kits were made available for the general public?*	1 = Very likely<br>2 = Somewhat likely<br>3 = Not likely at all<br>4 = Never again

Validation of self-testing with Ag-RDTs study

Observation Form_v1.0_26012022

1. Date *(dd/mm/yy)* _ _/_ _/_ _

2. Name of person filling-in the form: ______________________________

Steps	Correctly performed?	Comments (if no why not, what went wrong)
1. Opening of the packaging	Yes ☐<br>No ☐	
2. Opening the Buffer Bottle while keeping it vertically	Yes ☐<br>No ☐	
3. Squeezing liquid from Buffer Bottle into Tube	Yes ☐<br>No ☐	
4. Filling the Tube with liquid up or slightly above the fill line	Yes ☐<br>No ☐	
5. Placing the Tube in the rack	Yes ☐<br>No ☐	
6. Open swab protective package and taking swab out while keeping fingers out of swab end	Yes ☐<br>No ☐	
7. Inserting soft end of swab less than one inch (about 2 cm) into nostril	Yes ☐<br>No ☐	
8. Rotating the swab five times against the nasal wall.	Yes ☐<br>No ☐	
9. Using the same swab repeat collection procedure with the second nostril	Yes ☐<br>No ☐	
10. Inserting the swab into the Tube	Yes ☐<br>No ☐	
11. Swirling the swab tip inside the Tube, pushing into the wall of Tube at least five times	Yes ☐<br>No ☐	
12. Squeezing out the swab by squeezing the Tube with fingers	Yes ☐<br>No ☐	
13. Breaking the swab at the breakpoint and closing the cap of Tube	Yes ☐<br>No ☐	

14. Remove test device from package and place flat on surface	Yes ☐<br>No ☐	
15. Check liquid fin the Tube for bubbles	Yes ☐<br>No ☐	
16. Opening the white cap and dispensing 5 drops vertically on the device	Yes ☐<br>No ☐	
17. Do not move or touch device until the test is finished	Yes ☐<br>No ☐	
18. Reading result at 15 minutes.	Yes ☐<br>No ☐	
19. Has the participant completed the self-testing procedure?	Yes ☐<br>No ☐	
**Results section**<br>**(please check result on test device and recorded result on Participant Form)**		
20. What is the **result <u>on the test device</u>**?	Positive ☐<br>Negative ☐<br>Invalid ☐	
21. What is the **result recorded <u>on Participant's Form</u>**?	Positive ☐<br>Negative ☐<br>Invalid ☐	
22. Was the self-testing repeated?	Yes ☐<br>No ☐	
23. What is the **result <u>on the test device for repeated testing ?</u>**	Positive ☐<br>Negative ☐<br>Invalid ☐	
24. What is the **result recorded <u>on Participant's Form for repeated testing</u>**?	Positive ☐<br>Negative ☐<br>Invalid ☐	
