# Supplementary material for: Evaluation of COVID-19 antigen rapid diagnostic tests for self-testing in Lesotho and Zambia
Source: PLoS One. 2024 Feb 29;19(2):e0280105. doi: 10.1371/journal.pone.0280105 (PMC10903820; doi:10.1371/journal.pone.0280105)

S3 Appendix. Instructions for Use (IFU) for SARS-CoV-2 self-testing in Lesotho

MistraL

Procedure for SARS-CoV-2 Self Rapid Test using Nasal Swab

3. Run the rapid Antigen test

1. After removing the swab from the second nostril, insert it into the extraction buffer tube.

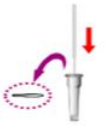

2. While squeezing the buffer tube, stir the swab more than 5 times.

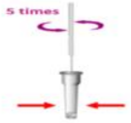

3. Remove the swab while squeezing the sides of the tube to extract the liquid from the swab. Discard the swab in an appropriated bin.

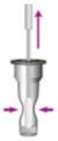

4. Press the nozzle cap tightly on the buffer tube.

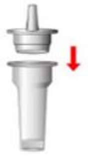

5. Apply 4 drops of the extracted specimen to the specimen well on the test device. Discard the tube in a bin, wash or sanitise your hands.

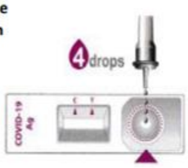

6. Read the test result in 15 to 30 minutes.

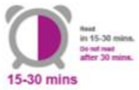

4. Interpret the Result

Positive

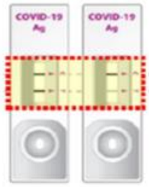

Negative

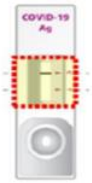

Invalid

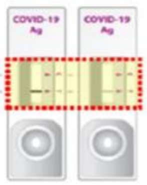

MistraL

Procedure for SARS-CoV-2 Self Rapid Test using Nasal Swab

1. Prepare the Testing kit

1. Check the expiry date on the back of the foil pouch. Do not use if the kit has expired.

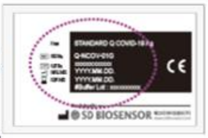

2. Open the foil pouch and check the desiccant. Do not touch the specimen well on the test device and only use the test kit if the content of the desiccant is green.

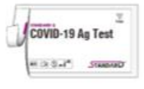

<Foil pouch>

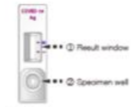

<Test device>

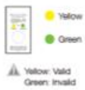

<Desiccant>

2. Collect the nasal swab

1. Wash or sanitise your hands. Open the swab pack and take the swab out without touching the soft end.

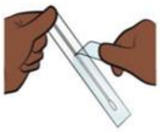

2. Tilt your head slightly. While rotating the swab, insert the swab for about 2cm (or until you feel resistance) in your nostril.

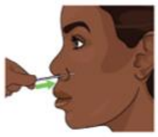

3. Rotate the swab 4 times against the nasal wall.

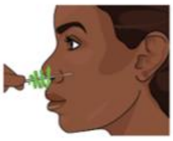

4. Gently remove the swab from the first nostril.

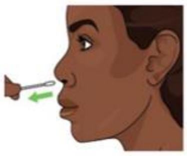

5. Insert the swab in the second nostril for about 2 cm (or until you feel resistance) and rotate 4 times against the nasal wall.

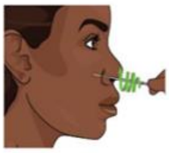

Supplement: S3 Appendix — (PDF) [file pone.0280105.s003.pdf]
