# Supplementary material for: Evaluation of COVID-19 antigen rapid diagnostic tests for self-testing in Lesotho and Zambia
Source: PLoS One. 2024 Feb 29;19(2):e0280105. doi: 10.1371/journal.pone.0280105 (PMC10903820; doi:10.1371/journal.pone.0280105)
Supplement: S4 Appendix — (PDF) [file pone.0280105.s004.pdf]

S4 Appendix. Definitions

Symptomatic:

In Zambia, participants were classified as symptomatic if they had any of the following COVID-19 symptoms: cough, fever, shortness of breath or loss of smell or taste, while in Lesotho participants were classified as symptomatic if they self-reported either cough, fever, shortness of breath, loss of taste or smell, pronounced tiredness, sore throat, muscle or body pain, diarrhea, weight loss, night sweat, chest pain, headache, rash on skin.

Usability:

Usability in this study is defined as a participant's ability to complete the test, and how easy or difficult it was to understand the instructions and complete each step in the process.

Acceptability:

Acceptability was measured in terms of people consenting to and using the provided self-test, and the proportion who reported they would be willing to repeat a self-test in the future.
