# Supplementary material for: Evaluation of COVID-19 antigen rapid diagnostic tests for self-testing in Lesotho and Zambia
Source: PLoS One. 2024 Feb 29;19(2):e0280105. doi: 10.1371/journal.pone.0280105 (PMC10903820; doi:10.1371/journal.pone.0280105)
Supplement: S5 Appendix — (PDF) [file pone.0280105.s005.pdf]

Cognitive results- country comparison

What was common to both IFUs:

1. Checking expiry date at the beginning and checking that there is a desiccant in the package before proceeding with the process
2. Washing or sanitizing one's hands, and not touching the tip of the swab as one opens the swab pouch
3. Process of collecting the specimen- including tilting the head at an angle and inserting swab into the nostrils for a specified distance, rotating the swab for a specific number of times
4. Process of testing the specimen, waiting for results, and interpreting the results

		**Lesotho**	**Zambia**
1	Understanding of both pictorial and written instructions / sufficiency of instructions for comprehension	Some of the participants got a bit confused on how many times they had to rotate the nasal swab in the nostril versus stirring the swab in the buffer<br><br>*"it's not that hard but I was a bit confused of how many times I have to rotate the swab in my nose versus stirring in the swab. But I think that's because this was my first experience"* [69 years, male participant].	1. Most participants relied on images to perform the instructions; did not understand the words nozzle, cap and pouch<br>2. An example in which most participants (3/5) found both the image and the written instructions hard to comprehend is phase 5 (setting the solution tube on the stand hole) and did not therefore perform the action. *"The package on the IFU is gray with a red stand hole while the actual package is red and white with a gray stand hole*". Others found a way of ensuring that the specimen tube stood upright- setting it against the box. This did not affect the overall testing process and outcome
2	Clarity/ understanding of the images<br>The watch.	The watch. The image of the watch was deemed not to be clear	The watch. The image of the watch was deemed not to be clear. *"For people to know it is 15 minutes, you need to put a watch with labels or handles and numbers"* [Participant 2].<br><br>*"For people to know it is 15 minutes, you need to put a watch with labels or handles and numbers"* [Participant 2]<br><br>Suggested for the watch on the instructions to have 'normal numbers'. And for the watch to be included on the list of items needed when testing in phase 2 [Participant 3]<br><br>Clustered images were difficult to comprehend (phase2, participant 1: Images were confusing, sterile swab package looked like a mirror & test device. - Package like a card. He identified the rest) (phase2, participant 2: thought there were 8 items. - Was able to identify the sterile swab due to its 'see through' packaging)
3	Use of difficult words, terms (comprehension of	They further demonstrated that there is enough understanding that they have to avoid	Nozzle cap, desiccant, foil pouch, specimen well, sterile swab, nostril,

	words and suggested words)	touching the part which is meant to go inside the nostril.	
4	Missing items from the test kits	Sanitizer- appreciate the importance of having sanitizer or washing hands before opening the swab pack: The community members added that they struggle to have sanitizers due to lack of affordability.<br><br>*"I think its important to clean our hands because we might have germs, and they ought to be killed before we start the work so that they don't interfere with the test results"* [21 years, male Participant]	1. Watch was not included- but critical to the testing process- or at least prompt the tester at the beginning that they will need a watch to time the test<br>2. Gloves not included
5	Critical information that should be easily and quickly identified:<br>1. Expiry date<br>2. Use of desiccant	1. Expiry date was quickly seen by everyone<br>2. Desiccant<br>3. Did not appreciate the importance of the preparation stage: while others thought the instructions for preparations were meant to have everything within reach so that some of the items don't fall-off as they start the testing procedures or end up forgetting other items, others were not aware of the significance of this instruction at all.<br>*"I think its important to have everything within reach to avoid other items falling of and getting contaminated when you try to reach them"* [34 years, female participant]	All **participants had a challenge with knowing the use of the desiccant** and majority could not find the expiry date on the foil pouch in step three
6	Use of local images/ items people are used to	Yellow = valid<br>Green = invalid	Yellow = valid<br>Green = invalid
7	Measurement	The instructions on the insertion of the swab in the nostrils are a bit unclear, they did not know how far 2 centimeters is.<br>*"It was a bit hard to know how deep 2 centimeters is"* (21 years, female participant].	

8	Squeeze	Did	Did not – only 1/5
9	Interpreting the results	1. All/ most of the participants correctly interpreted the results? ..but struggled to differentiate from the negative and the invalid test results.<br>*"Since the two tests look similar, it would really be hard to differentiate between a negative and an invalid test. The lines are a bit blur, it's what to say what is really going on"* [63 years, male participant].<br>2. General recommendation was that those who can't read will not understand the word invalid	1. All the participants correctly interpreted the results<br>2. Although the term invalid was not understood by all participants …..one knew it meant retest<br>3. General recommendation was that those who cant read will not understand the word invalid

1. Overall, participants performed the test with some not necessarily needing to complete all the steps successfully.
2. Interpreting the results: Was the only step for which written instructions were critical. The word 'positive' is a commonly used one and that helped in the interpretation of the test results.

Cognitive Interview response matrix (Biosensor)- Zambia

	Participant no, age, gender	Level of education	Comprehension of words, and suggested words	Clarity of image and what it depicts, and suggested image (s)	Performing action (easy/ difficult)	Words or images (what tester relied on more to perform the action)	Would other people understand words, images?	Other observations and suggestions
	**Step 1: Preparation:**							
Phase1: Preparation	Partic 1: Male, 20years	Grade 10	Easily comprehended the words.	The image was clear.	Easy to perform (washed his hands in a dish).	Both words and images.	- Others will easily do this.<br>- Some might overlook the instruction on 'wearing gloves'.<br>- People can't afford gloves.	Put instruction on 'wearing gloves' on top before the image.
	Partic 2: Female, 66years	Form 3	Easily comprehended the words.	The image was clear.	Did not wash hands, thought hands were clean.	Both words and images.	- Those who can't read won't know they need to wear gloves.<br>- People can't afford gloves.<br>- Some won't wash their hands before testing	- Put instruction on 'wearing gloves' on top before the image.<br>- Instructions to be written in local languages.<br>- Free gloves to be included in kits
	Partic 3: Female, 19years	Grade 12	The participant partially comprehended the words. She thought wearing of gloves was an alternative in case one didn't wash their hands.	The image was clear.	Easily performed the action.	Both words and images.	-Others will easily do it due to availability of water.<br>-Others won't afford gloves.	"the instructions should only advise people to wash their hands as some won't

								be able to afford gloves”.
	Partic 4: Male, 32years	College (Seemed to be lying).	Easily comprehended the words.	Image was very clear.	Easily performed action.	Relied on both words and image.	Those who can’t read won’t know they need to wear gloves.	“The gloves are the challenge because they are mostly found in clinics, you need to pack the gloves in the packaging”.
	Partic 5: Male, 58years	Grade 9	Easily comprehended the words.	Image was clear.	Easily performed action.	Relied on both words and image.	Others will understand this.	Since people won’t afford gloves, they could use the small transparent plastic bags as an alternative.
Phase2: Checking the contents	Partic 1:		Did not fully comprehend the instruction.	- Images were confusing, sterile swab package looked like a mirror & test device.<br>- Package like a card.<br>- He identified the rest.	Only identified the test device, solution tube and nozzle cap, and the instruction menu & wondered what the rest were.	Both words and images.	People will have challenges in knowing the images are showing the content & packaging.	
	Partic 2:		Had challenges in comprehending some words, “I don’t understand words like nozzle and swab, I don’t	- Images were confusing, thought there were 8 items.<br>- Was able to identify the sterile swab due	-Had a lot of difficulty and almost gave up due to the confusing images.	Both the images and words were very confusing.	Only the educated & those who have been to COVID-19 testing workshops will understand this.	-Contents should be drawn on top of the packaging so that people are able to

			understand this English".	to its 'see through' packaging.	-Managed to identify the test device, solution tube and nozzle cap after removing them from the packaging.			see what is inside.<br>-Instructions should be brief & in local languages.
	Partic 3:		Was able to comprehend the words.	Images were very clear.	Easily performed the action.	Relied more on words than images.	Others will easily understand this.	
	Partic 4:		Failed to understand the terms 'nozzle cap', 'check the kit content' and 'instruction menu'.	Images were clear.	Easy for the participant to perform action.	Relied on images.	Other people would understand this.	
	Partic 5:		Was able to comprehend the words.	The images were clear but had some initial challenges in identifying the instruction menu.	Easily performed the action.	Relied more on words.	People who can't read might find this challenging.	
Phase3: Checking expiry date, checking the devices	Partic 1:		Did not understand the words 'desiccant' and 'foil pouch'.	Challenges with understanding the desiccant image & what it meant.	-Could not locate the expiry date as "it was not visible".<br>-Thought the desiccant was to be used for testing by pouring its content on the test device. "Yellow meaning results are bad and	Used both words and images.	People might have challenges in finding the expiry date and knowing the use of the desiccant.	

					Green are good”.			
	Partic 2:		-Only understood the term ‘results window’.<br>-Used the word ‘expiry’ to know she had to check for an expiry date but didn’t know for what.<br>-Did not understand the term ‘specimen well’.<br>-Did not understand what the words valid & invalid meant. Thought it meant positive & negative result.	-Images were not clear.<br>-Difficult to see expiry date on the image.	-Participant got stuck in this phase for some time.<br>-She took all items out of the packaging & started guessing what to do.	Barely relied on both words and images. Did not understand them.	Only the educated and people who have attended COVID-19 testing workshops can understand this.	-“Put the date exactly after ‘exp date’ here”.<br>-For the desiccant “words should be in local languages; Green – expired and Yellow- active or working”.
	Partic 3:		The participant did not understand the word ‘desiccant’.	Images were clear.	-Took some time to locate the expiry date. Had some challenges.<br>-Did not remove the desiccant from the foil pouch as she did not see it.	Used both words and images.	Others will understand this	
	Partic 4:		Could not comprehend the words.	Images were not clear. He could not understand that he had to check the expiry date on the foil	Participant failed to complete the testing process.	Failed to understand both words and images.		

				pouch. He did not understand the use of the desiccant.				
	Partic 5:		Participant did not understand the word desiccant.	Images were clear.	-Participant checked the expiry date on the kit instead of the foil pouch.<br>-He did not understand the use of the desiccant and did not know it was in the foil pouch. He only saw it after the testing process when he was told by the social scientist.	Relied on both words and images.	Some people will be able to understand this.	
	**Step 2: Sample Collection:**							
Phase4: Opening the solution tube and nozzle cap	Partic 1:		Did not understand the term ‘nozzle cap pouch’.	Images were very clear.	Had a challenge in identifying what the nozzle cap was for in this phase.<br>But did not face any challenge when performing the action.	Mostly depended on images.	“Other people will easily understand this step”.	
	Partic 2:		Could not comprehend the words.	Had a challenge in seeing that the hands on the image were removing the	It took some time for the participant to realize she had to remove the	Depended on the images.	Only the educated and those who have attended COVID-19 testing	

				solution tube seal.	solution tube seal.		workshops will easily figure this out.	
	Partic 3:		Was able to comprehend the words.	Images were clear.	Easily performed the action.	Mostly relied on images.	Others will understand the instruction.	
	Partic 4:							
	Partic 5:		Did not comprehend the words.	Images were clear. "Everything is fine because the picture has helped".	Easily performed the action.	Relied on images.	Others will understand this.	
Phase5: Setting the solution tube on the stand hole	Partic 1:		Could not comprehend the words.	The image was very confusing, "The package on the IFU is gray with a red stand hole while the actual package is red and white with a gray stand hole".	Did not manage to perform the action.	Both words and images were not helpful.	People will find this challenging.	
	Partic 2:		Could not comprehend the words.	The image was confusing.	Did not manage to perform the action.	Both words and images were not helpful.	People will find this challenging.	"For the stand hole maybe if its function was written on the box in a local language".
	Partic 3:		Easily comprehended the words.	The image was clear.	Easily performed the action.	Used both words and images.	Others will understand the instruction.	
	Partic 4:							
	Partic 5:		Was not able to comprehend the words.	The image was confusing.	Unable to perform action. "Fix the solution tube, how will I	Both words and images were confusing.	Others will have challenges.	

					fix it? This (nozzle cap) how to fix it".			
Phase6: Opening the sterile swab pouch	Partic 1:		Term 'sterile swab' difficult to understand.	Images were easy to understand, "the arrows pointing on the opposite sides mean that I have to open this (swab pouch), the X on the image mean not to hold the tip of the swab".	Easy for participant to perform but did touch the tip of the sterile swab.	Relied on images.	Some people will easily understand this.	
	Partic 2:		Did not comprehend the words.	The image was easy for the participant to understand.	Easy to perform action.	Relied on images.	Some people will understand this.	Emphasis on workshops.
	Partic 3:		Easily comprehended the words.	Image was clear. "It is telling me to open the package and not to hold this side (swab tip), but this other side".	Easy to perform action.	Relied on both words and images.	Others will understand the instruction.	
	Partic 4:							
	Partic 5:		Unable to comprehend the words. . "The words are difficult to understand".	Images were clear.	Easily performed action.	Relied on the images to understand the instruction.	Others will understand this.	
Phase7: Inserting the sterile swab in the nostrils	Partic 1:		Was able to comprehend the words.	Images were clear, "the arrows in the nostrils indicate that one must rotate the swab".	Easily performed the action.	Relied on both words and images.	Other people will understand this phase.	

	Partic 2:		Did not understand the word ‘nostril’ and ‘sterile’. She also mentioned that she did not understand what ‘less than one inch’ meant.	Partially understood what the image meant. Image was confusing as it “looked like there were three sterile swabs”. Did not understand it meant rotating the swab & not inserting 10times.	The participant only inserted the sterile swab once in each nostrils and did not rotate the swab 10times in each nostril.	Relied on the image.	Only the educated and those attending COVID-19 testing workshops will understand this phase.	-“They should put a hand twisting the swab with arrows near the hand”<br>-Need for instructions in local languages.
	Partic 3:		Easily comprehended the instruction. “So I am supposed to rotate ten times this side and this side. I used words to understand this”.	Images were clear.	-Had challenges in estimating the 1.5cm depth.<br>-Only rotated the swab stick 10times as it was uncomfortable. “This is uncomfortable”.	Relied on words.	Others will understand this.	
	Partic 4:							
	Partic 5:		Unable to comprehend the words.	Images were clear.	Easily performed action.	Relied on images.	Others will understand this.	
	**Step 3: Test Procedure:**							
Phase8: Inserting the swab into the solution	Partic 1:		Could not fully comprehend the instructions.	Images were clear.	Did not squeeze the solution tube while stirring the swab and when removing it.	Mostly relied on the images.	Other people will easily understand this phase.	
	Partic 2:		Could not comprehend the instructions but understood what	Images were clear.	Did not squeeze the solution tube when stirring the	Relied on the image.	The educated and those attending COVID-19 testing	

			the disclaimer at the bottom of the images was asking her to do.		swab and when removing it.		workshops will understand this phase.	
	Partic 3:		Was able to comprehend the words.	Image was clear. “The image is showing that I have to rotate but not squeeze, other people who can’t read won’t know. You need to change this image”.	Easily performed the instruction.	Relied more on words than image.	People who can’t read won’t understand this step.	She suggested the need to change the image so that image so that people who can’t read will know that one has to squeeze the solution tube whilst stirring the swab
	Partic 4:							
	Partic 5:		Unable to comprehend words.	Images were partially clear.	Was not easy for the participant to perform the action. (Did not squeeze the solution tube whilst stirring the swab. He broke the swab in half and left the other half in the solution tube and he sealed it with the nozzle cap).	Relied on images.	Others will understand this.	
Phase9: Pressing the nozzle cap tightly onto the tube	Partic 1:		Participant was able to comprehend the instructions.	Images where very clear.	Easy to perform the action.	Mostly relied on the image.	Other people will easily understand this phase.	

	Partic 2:		Did not comprehend the words.	Images where very clear.	Easy to perform the action.	Relied on images.	Other people will understand this.	
	Partic 3:		Comprehended the words.	Images were clear.	Easily performed the action.	Mostly relied on words.	Others will understand this.	
	Partic 4:							
	Partic 5:		Unable to comprehend the words.	Images were clear.	Easily performed action.	Relied on images.	Others will understand this.	
Phase10: Applying 4 drops of the extracted sample	Partic 1:		Did not understand the term 'extracted sample'. He assumed it was the solution tube.	Image was clear. "The two arrows on the image made me understand that I was supposed to squeeze the tube".	Easily performed the action.	Both words and images.	It will be easy for people to understand this phase.	
	Partic 2:		Did not comprehend the words.	Images were very clear. "I used the picture to know I had to squeeze four drops".	Managed to perform the action. Though initially thought she had to prick the specimen well with the nozzle cap.	Relied on the images.	"Here some people will think one needs to prick the specimen well with the nozzle cap and not squeeze four drops like I initially did".	
	Partic 3:		The instruction at the bottom of the image was confusing. "I don't understand that".	Image was clear.	Performed the action.	Relied on both words and images.	Others will understand this.	"All the instructions at the bottom of the images should come before the images as they are confusing".

	Partic 4:							
	Partic 5:		Unable to comprehend the words.	Image was clear.	Performed action.	Relied on image.	Others will understand this.	
Phase11: reading the test results	Partic 1:		Participant was able to comprehend the instructions.	Images where very clear and he loved how the clock showed the 15minutes.	Easy to perform the action.	Relied on both image and words.	Other people will easily understand this phase.	
	Partic 2:		Participant was able to comprehend the words. “I read the words in order to know that I needed to wait for 15minutes”.	Image was not clear. She initially thought the watch was a machine containing the specimen in it (red part).	Was not easy to perform the action (had no watch).	Relied on words.	Those who can’t read won’t understand this phase.	“For people to know it is 15minutes, you need to put a watch with labels or handles and numbers”.
	Partic 3:		Comprehended the words.	Image was not clear. “There is need to have a normal watch with numbers”.	Easily performed the action.	Relied on words.	Those who can’t read won’t understand this.	Suggested for the watch on the instructions to have ‘normal numbers’. And for the watch to be included on the list of items needed when testing in phase 2.
	Partic 4:							
	Partic 5:		Comprehended the words.	Image was clear.	Easily performed action.	Relied on both words and images.	Others will understand this.	
	Step 4: Interpretation of tests result							

Negative; Positive; Invalid	Partic 1:		Did not understand what the term 'invalid meant'.	Images were clear.	Easy for the participant to perform the action.	Relied on both images and words.	It will be easy for other people to understand this step.	
	Partic 2:		Partially comprehended the words. "Negative means you are okay, positive meaning someone has the disease and invalid meaning to retest. I read saying it means you need a new sample"	The images were partially clear. "Some people can't know this by looking when it is empty like this (invalid image), does it mean they (results) are okay. I don't understand this, it is hard".	It was easy for the participant to read her results.	Relied on both words and images.	It will be difficult for some people to read their results by just looking at the images.	
	Partic 3:		Easily comprehended the words.	Partially clear. "Those who can't read won't know. Maybe if the test device had N and P (for negative and positive) than C and T".	Easily performed the action.	Relied on both words and images	Those who can't read will face challenges. "Those who can't read won't know what invalid and valid results are"	
	Partic 4:							
	Partic 5:		Comprehended the words. "Invalid means you need to redo the test".	Images were clear.	Easily performed action.	Relied on both words and images.	Others will understand this. "Those who can't read will be able to know their results because of C and T".	

General Suggestions

- Instructions at the bottom of the images should be put at the top.

- Instruction menus to be written in local languages in order to be inclusive.
- A watch should be included as one of the items needed when testing.
- Kits should come with plastic gloves.
